# Evaluation of Between-County Disparities in Premature Mortality Due to Stroke in the US

**DOI:** 10.1001/jamanetworkopen.2021.4488

**Published:** 2021-05-12

**Authors:** Suhang Song, Gaoting Ma, Michael G. Trisolini, Kenneth A. Labresh, Sidney C. Smith, Yinzi Jin, Zhi-Jie Zheng

**Affiliations:** 1 Taub Institute for Research in Alzheimer’s Disease and the Aging Brain, Columbia University, New York, New York; 2 Department of Interventional Neuroradiology, Beijing Tiantan Hospital, Affiliated with Capital Medical University, Beijing, China; 3 RTI International, Research Triangle Park, North Carolina; 4 Division of Cardiology, School of Medicine, University of North Carolina at Chapel Hill; 5 Department of Global Health, School of Public Health, Peking University, Beijing, China; 6 Institute for Global Health, Peking University, Beijing, China

## Abstract

**Importance:**

Identifying the factors associated with premature stroke mortality and measuring between-county disparities may provide insight into how to reduce variations and achieve more equitable health outcomes.

**Objective:**

To examine the between-county disparities in premature stroke mortality in the US, investigate county-level factors associated with mortality, and describe differences in mortality disparities by place of death and stroke subtype.

**Design, Setting, and Participants:**

This retrospective cross-sectional study linked the mortality and demographic data of US counties from the Centers for Disease Control and Prevention WONDER database to county-level characteristics from multiple databases. The outcome measure was county-level age-adjusted stroke mortality among adults aged 25 to 64 years in 2637 US counties from 1999 to 2018. This study was conducted from April 1, 2019, to October 31, 2020. Generalized linear Poisson regressions were fitted to investigate 4 sets of factors associated with county-level mortality: demographic composition, socioeconomic status, health care and environmental features, and population health. The Theil index score was calculated to assess the mortality disparities.

**Main Outcomes and Measures:**

Stroke mortality was measured as the number of deaths attributed to stroke in the data set. Out-of-stroke-unit death was defined as any death occurring in outpatient or emergency departments or at the pretransport location. Five stroke subtypes were included in the analysis.

**Results:**

Although mortality did not change substantially from 1999 to 2018 (from 12.62 to 11.81 per 100 000 population), the proportion of deaths occurring out of the stroke unit increased from 23.56% (4328 of 18 369) to 34.57% (6978 of 20 188). A large percentage of stroke of an uncertain cause was reported, with most deaths (55.20%) occurring out of the stroke unit. In the county with the highest premature stroke mortality, the incidence was 20.78 times as high as that in the county with the lowest mortality (65.04 vs 3.13 deaths per 100 000 population). The highest between-county disparities were found for stroke of uncertain cause. For out-of-stroke-unit death, county-level mortality was largely associated with demographic composition (31.6%) and health care and environmental features (25.8%). For in-hospital death, 29.8% of county-level mortality was associated with population health and 28.7% was associated with demographic composition.

**Conclusions and Relevance:**

These findings suggest that strategies addressing specific factors that underlie the mortality disparities among US counties, especially for out-of-stroke-unit death and stroke of uncertain cause, may be useful when tailored to the county-level context before implementing interventions for the neediest counties.

## Key Points

**Question:**

Are demographic composition, socioeconomic status, health care and environmental features, and population health associated with premature stroke mortality?

**Findings:**

In this cross-sectional study examining the differences in premature stroke mortality in terms of between-county disparity in the US, for deaths that occurred out of the stroke unit, county-level mortality was largely associated with demographic composition (31.6%) and health care and environmental features (25.8%). For in-hospital death, 29.8% of county-level mortality was associated with population health and 28.7% was associated with demographic composition.

**Meaning:**

These findings suggest the need to tailor strategies to address premature stroke in the county-level context before implementing interventions for the neediest counties.

## Teaser

This cross-sectional study compares the numbers and factors associated with death due to stroke in younger and middle-aged adults among US counties.

## Introduction

The occurrence of stroke in younger and middle-aged adults has a serious socioeconomic impact and can lead to more lost productivity than stroke occurring later in life.^1^ Patterns in premature stroke mortality may serve as a sentinel for identifying adverse changes and may aid in targeting interventions. Although a decline in mortality due to stroke has been noted in the US over the past decades, there remain substantial geographic disparities in mortality, with higher rates in the southeastern US, termed the “stroke belt.”^2^

However, most studies have reported on the stroke mortality and its disparity by state.^3^ County-level data are valuable because many public health programs and policies are designed and implemented at this local level. Moreover, few studies examined how premature stroke mortality is associated with county-level demographic and socioeconomic data, health risk profiles, or access to quality health care. Identifying such factors that are associated with between-county mortality may provide insight into how to reduce disparities and achieve more equitable health outcomes.

Furthermore, successful acute stroke intervention depends on early recognition of symptoms, prompt emergency transport, and rapid in-hospital treatment. Approximately 25% to 27% of premature stroke deaths occur before admission to the hospital, and the place of death varies by stroke subtype.^4^ For example, acute stroke of a sudden and unexpected nature occurring out of a stroke unit may be associated with continuing problems of delays in seeking care.

Large differences across counties in out-of-hospital mortality more likely reflect the differences in population characteristics, health risk profile, or health care quality; large differences in in-hospital county-level mortality might suggest major differences in health status and health care delivery. However, no study, to our knowledge, has assessed the comparability of mortality disparities by place of death and stroke subtype across US counties.

To address this gap, this study aimed to examine between-county disparity in premature mortality due to stroke in the US, investigate county-level factors associated with mortality, and illustrate differences in mortality disparities by place of death and stroke subtype. The findings of this study may provide information for better interpretation of mortality by place of death and stroke subtype and assist in the designing of interventions for the neediest clusters of counties.

## Methods

### Study Population and Data

This cross-sectional study defined stroke death as death for which the underlying cause was categorized in the *International Statistical Classification of Diseases and Related Health Problems, Tenth Revision* as subarachnoid hemorrhage (code I60), intracerebral hemorrhage (codes I61 and I62), cerebral infarction (code I63), stroke not specified as hemorrhage or infarction (code I64), other cerebrovascular disease (code I67), and sequelae of cerebrovascular disease (code I69).^5^ Stroke mortality was measured as the number of deaths attributed to stroke in the data set. We defined out-of-stroke-unit death as any death occurring in the outpatient or emergency department; when a patient is pronounced dead on arrival; or death at a pretransport location, including the decedent's home, hospice facility, nursing home, or long-term care facility; and defined in-hospital death as a death occurring within inpatient facilities.^4,6^ We also defined a third group including deaths occurring in other places, place of death unknown, or status unknown. All analyses were restricted to individuals aged 25 to 64 years to focus on premature death.^7,8^

This study, conducted from April 1, 2019, to October 31, 2020, analyzed the US mortality data during 1999-2018 using mortality and demographic variables from death certificates from the US National Center for Health Statistics of the Centers for Disease Control and Prevention (the CDC WONDER database).^9^ We included the following data: age, sex, race, underlying cause of death, place of death, county, and year. Race was ascertained from death certificates and classified as White, Black or African American, American Indian or Alaskan Native, and Asian or Pacific Islander; sex was classified as male and female, and these data were also obtained from the CDC WONDER database. Because the database does not provide the number of deaths if the death count is less than 10 based on the database guideline, counties with these low numbers were combined to create stable units of analysis. During 1999-2018, the databases provided reliable data on 2637 counties.^9^ This study used publicly available data and was deemed exempt from guidelines for human research by the Peking University Institutional Review Board. This study followed the Strengthening the Reporting of Observational Studies in Epidemiology (STROBE) reporting guideline.

We used county codes to link the mortality and demographic data of each county to the county-level characteristics from 6 databases in 2011-2018, which had been introduced in some earlier studies.^10,11^ During 2011-2018, 2050 counties were included for analysis. The combined data set comprised variables associated with stroke mortality at the county level. According to the previous studies, 4 sets of county-level factors included demographic composition, socioeconomic status, health care and environmental features (eg, primary care physicians per 100 000 population, and health care quality index), and population health (eg, physical inactivity rate, self-reported poor health status).^10,11,12^ eTable 1 in the Supplement provides data sources and summary statistics for each of these variables.

### Statistical Analysis

The unit of analysis was county level. We assessed age-adjusted mortality by place of death for groups defined by sex, age group, race, and cause of death. The Theil index, a measurement of relative disparity between counties, was used to assess the disparity in premature stroke mortality. The calculation is based on the proportion of the population in each county and the ratio of the mortality in each county to the overall mortality in the entire population. The Theil index is a positive value with no maximum level, and a score of 0 indicates absolute equality. The higher the index score, the larger disparity in mortality among counties.^13^ The advantage of the Theil index is that it can break down the overall disparity into within- and between-state categories.^14,15,16^ Overall disparity can be estimated with the county-level mortality in all counties in the US. Within-state disparity can be estimated with the counties in a particular state. Between-state disparity is the difference between the overall disparity and within-state disparity (overall Theil index score minus within-state Theil index score). We also examined the between- and within-state disparity by place of death and stroke subtype. Age-adjusted mortalities were standardized by the direct method to the standard US population in 2000.

We conducted generalized linear Poisson regressions to estimate the associations between county-level factors and premature stroke mortality; all factors were included in the model simultaneously. To quantify the extent to which the 4 sets of factors were associated with the premature stroke mortality, we conducted the dominance analysis for decomposition by examining the relative importance of these variables. The dominance statistics can be used as an index of effect size.^17^ This procedure is an examination of the *R*^2^ for all possible subset models of the regression.^18,19^ The final model was also conducted for groups by place of death and stroke subtype. All analyses were conducted in Stata, version 14.1 (StataCorp LLC). Two-sided testing at *P* < .05 indicated significance.

## Results

During 1999-2018, 385 831 stroke deaths occurred among adults aged 25 to 64 years, among which 170 577 deaths (10.53 per 100 000 population) were in women. The age-adjusted rate was 12.04 per 100 000 population, of which 27.96% occurred out of a stroke unit (107 878 of 385 831) and 69.26% occurred in the hospital (267 211 of 385 831) (Table 1). Although mortality rates did not change substantially (from 12.62 to 11.81 per 100 000 population) from 1999 to 2018, the proportion of deaths due to stroke occurring out of the stroke unit increased from 23.56% (4328 of 18 369) to 34.57% (6978 of 20 188) (eFigure in the Supplement). The leading cause of death was intracerebral hemorrhage, with a rate of 4.29 per 100 000 population, and a rate of 3.80 for deaths not specified as caused by hemorrhage or infarction. The highest proportion of deaths occurring out of a stroke unit was 55.20% for other and sequelae of cerebrovascular disease (21 998 of 39 857) compared with 15.18% for intracerebral hemorrhage (20 837 of 137 279) (Table 1).

**Table 1.** Mortality Due to Stroke and Distribution of Place of Death and Subtype in US Residents, 1999-2018

Variable	No. of deaths (death rate per 100 000)	No. of deaths (%)^a^							
		Out-of-stroke-unit						In-hospital	Other, place or status unknown
		Dead on arrival	Outpatient/emergency department	Decedent's home	Hospice facility	Nursing home/long-term care	Total		
Total	385 831 (12.04)	1838 (0.48)	23 371 (6.06)	38 438 (9.96)	12 382 (3.21)	31 849 (8.25)	107 878 (27.96)	267 211 (69.26)	10 742 (2.78)
Sex									
Male	215 254 (13.59)	1074 (0.50)	12 827 (5.96)	22 143 (10.29)	6943 (3.23)	18 159 (8.44)	61 146 (28.4)	147 588 (68.56)	6520 (3.02)
Female	170 577 (10.53)	764 (0.45)	10 544 (6.18)	16 295 (9.55)	5439 (3.19)	13 690 (8.03)	46 732 (27.40)	119 623 (70.13)	4222 (2.47)
Age group, y									
25-34	11 125 (1.34)	97 (0.87)	1047 (9.41)	1126 (10.12)	123 (1.11)	250 (2.25)	2643 (23.76)	8155 (73.30)	327 (2.94)
35-44	41 413 (4.89)	349 (0.84)	3458 (8.35)	4065 (9.82)	611 (1.48)	1304 (3.15)	9787 (23.64)	30 429 (73.48)	1197 (2.88)
45-54	116 258 (13.74)	614 (0.53)	7488 (6.44)	11 157 (9.60)	2844 (2.45)	6339 (5.45)	28 442 (24.47)	84 395 (72.59)	3421 (2.94)
55->64	217 035 (31.84)	778 (0.36)	11 378 (5.24)	22 090 (10.18)	8804 (4.06)	23 956 (11.04)	67 006 (30.88)	144 232 (66.46)	5797 (2.66)
Race									
White	263 181 (10.26)	1162 (0.44)	14 726 (5.60)	28 833 (10.96)	9018 (3.43)	21 458 (8.15)	75 197 (28.58)	180 961 (68.76)	7023 (2.66)
Black or African American	103 108 (24.86)	604 (0.59)	7601 (7.37)	8052 (7.81)	3001 (2.91)	9243 (8.96)	28 501 (27.64)	71 318 (69.17)	3289 (3.19)
American Indian or Alaska Native	3335 (8.57)	10 (0.30)	170 (5.10)	310 (9.30)	100 (3.00)	249 (7.47)	839 (25.17)	2390 (71.66)	106 (3.17)
Asian or Pacific Islander	16 207 (8.77)	62 (0.38)	874 (5.39)	1243 (7.67)	263 (1.62)	899 (5.55)	3341 (20.61)	12 542 (77.39)	324 (2.00)
Cause of death									
Subarachnoid hemorrhage	59 865 (1.87)	522 (0.87)	5491 (9.17)	7018 (11.72)	661 (1.10)	914 (1.53)	14 606 (24.39)	43 307 (72.34)	1952 (3.27)
Intracerebral hemorrhage	137 279 (4.29)	397 (0.29)	7742 (5.64)	7037 (5.13)	2628 (1.91)	3033 (2.21)	20 837 (15.18)	113 388 (82.60)	3054 (2.22)
Cerebral infarction	27 095 (0.85)	86 (0.32)	891 (3.29)	2073 (7.65)	1264 (4.67)	1760 (6.50)	6074 (22.43)	20 409 (75.32)	612 (2.25)
Stroke, not specified as hemorrhage or infarction	121 735 (3.80)	590 (0.48)	6423 (5.28)	14 727 (12.10)	5807 (4.77)	16 816 (13.81)	44 363 (36.44)	73 548 (60.42)	3824 (3.14)
Other and sequelae of cerebrovascular disease	39 857 (1.24)	243 (0.61)	2824 (7.09)	7583 (19.03)	2022 (5.07)	9326 (23.40)	21 998 (55.20)	16 559 (41.55)	1300 (3.25)

^a^ The percentages presented are row percentages within the category.

### Between-County Disparity

**Figure 1.**
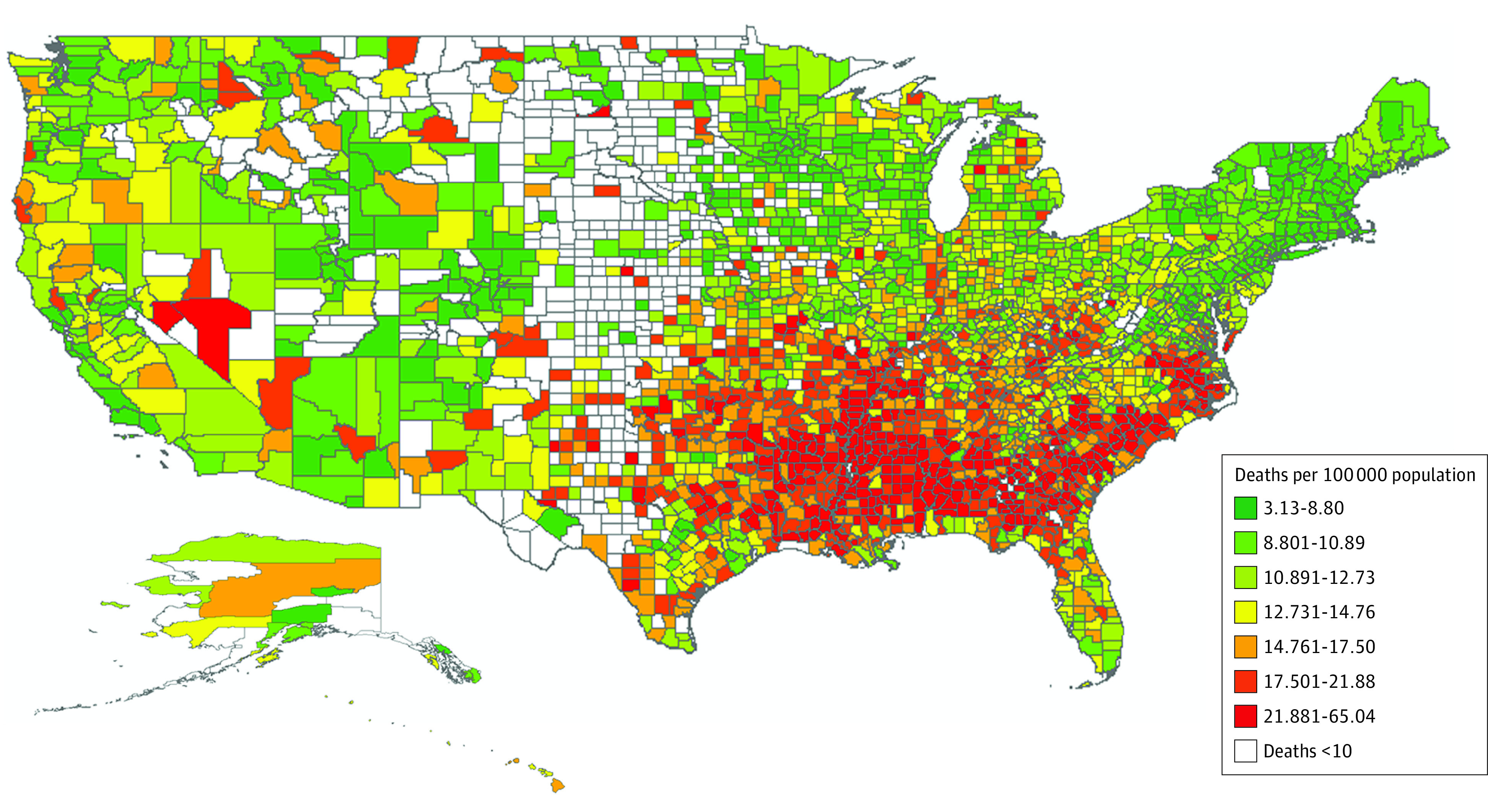
County-Level Crude Mortality Due to Stroke in US Residents Aged 25 to 64 Years, 1999-2018 Mortality was calculated as the number of deaths per 100 000 population. The map contours were obtained from the US Census Bureau, 2018 US County Cartographic Boundary Files–Shapefile. The analysis was performed with platform ArcGIS (esri).

**Figure 2.**
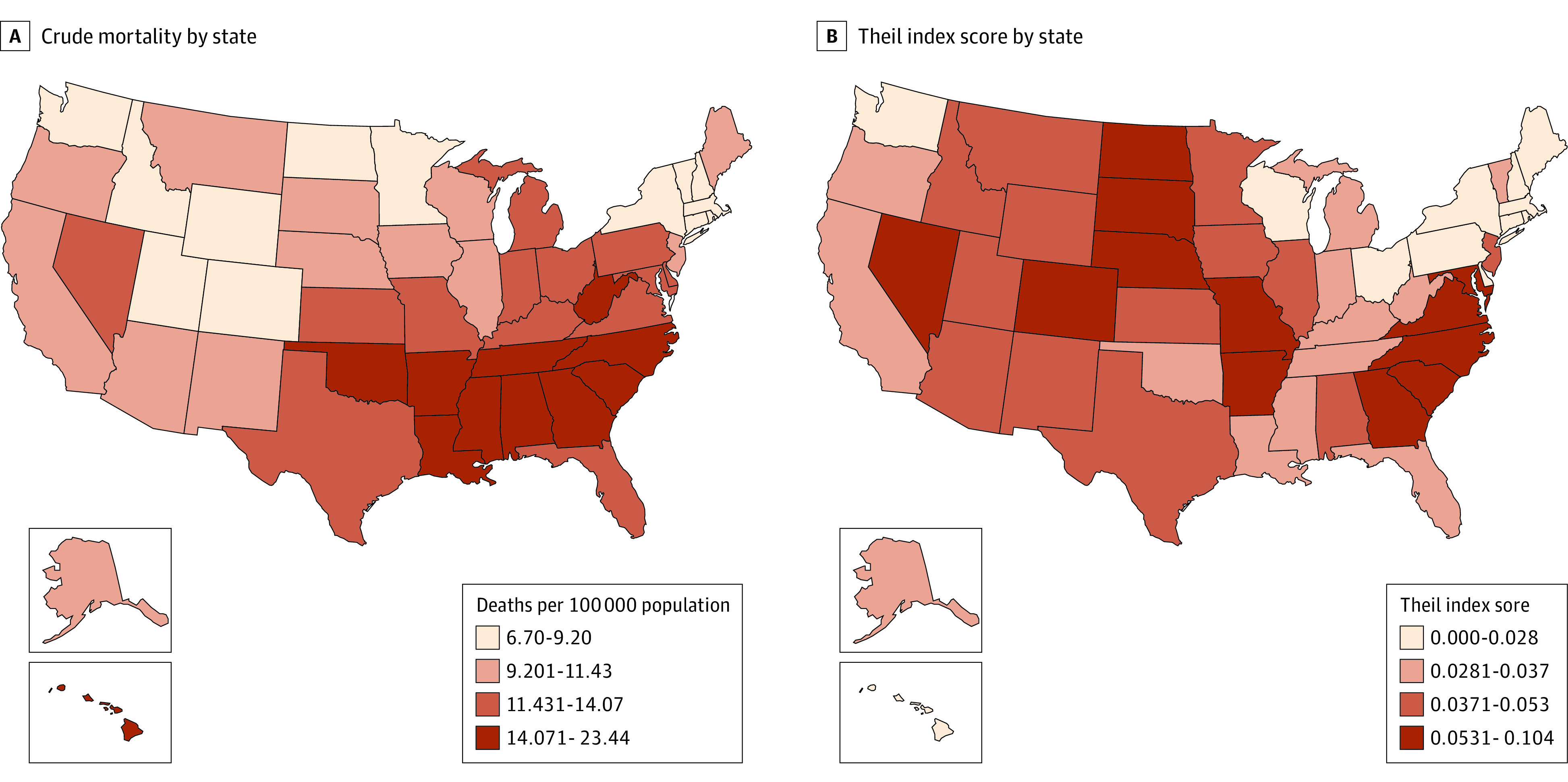
Between and Within-State Disparity in Stroke Mortality in US Residents Aged 25 to 64 Years, 1999-2018 A, Crude mortality rate by state. B, Thiel index score by state. Mortality was calculated as the number of deaths per 100 000 population.

The Theil index was 0.091 for county-level mortality and was broken down by between-state and within-state variation, with within-state variation accounting for 51.5% (within-state Theil index score, 0.047; overall Theil index score, 0.091) of the overall disparity (Table 2). The mortality rate was variable across counties—the county with the highest mortality was 20.78 times as high as that with the lowest mortality (65.04 vs 3.13 deaths per 100 000 population) during 1999-2018. The counties with the highest mortality were in the southeastern stroke belt band, stretching from the Ohio River Valley to the Mississippi River Valley (Figure 1). The variation was evident within each state, including states with the lowest mortality. For example, Colorado had a relatively low mortality rate at 8.07 per 100 000 population, which was the seventh lowest of 50 states and the District of Columbia, but had a relatively high within-state disparity in mortality (Theil index score, 0.085), which was the fifth highest state (Figure 2).

**Table 2.** Between-County Disparities in Mortality Due to Stroke and by Place of Death and Subtype in US Residents 1999-2018

Variable	Overall	Within-state (contribution, %)	Between-state (contribution, %)
Death rate per 100 000	0.091	0.047 (51.5)	0.044 (48.5)
Place of death, %			
Out-of-stroke-unit	0.135	0.077 (57.0)	0.058 (43.0)
Dead on arrival	0.566	0.125 (22.2)	0.440 (77.8)
Outpatient/emergency department	0.236	0.111 (47.1)	0.125 (52.9)
Decedent's home	0.255	0.144 (56.2)	0.112 (43.8)
Hospice facility	0.277	0.133 (48.2)	0.144 (51.8)
Nursing home/long-term care	0.185	0.118 (64.1)	0.066 (35.9)
In-hospital	0.092	0.050 (54.5)	0.042 (45.5)
Other, place or status unknown	0.474	0.124 (26.2)	0.350 (73.8)
Cause of death, %			
Subarachnoid hemorrhage	0.055	0.046 (82.8)	0.009 (17.2)
Intracerebral hemorrhage	0.107	0.060 (55.8)	0.047 (44.2)
Cerebral infarction	0.151	0.097 (64.3)	0.054 (35.7)
Stroke, not specified as hemorrhage or infarction	0.169	0.083 (49.4)	0.085 (50.6)
Other and sequelae of cerebrovascular disease	0.171	0.114 (66.5)	0.057 (33.5)

Between-county disparities varied substantially by place of death and stroke subtype. The Theil index score for out-of-stroke-unit death was 0.135, which was higher than that for in-hospital death at 0.092. For out-of-stroke-unit death, within-state variation accounted for 57.0% (Theil index score, 0.077) of the overall disparity (Theil index score, 0.135). The highest between-county disparities were found for other and sequelae of cerebrovascular disease (Theil index score, 0.171) and those not specified as hemorrhage or infarction (Theil index score, 0.169), of which within-state variation accounted for 66.5% (Theil index score, 0.114 per 0.171) of strokes due to other and sequelae of cerebrovascular disease and 49.4% (Theil index score, 0.083 per 0.169) of strokes not specified as hemorrhage or infarction of the overall disparities (Table 2).

### Factors Associated With Premature Mortality

Dominance analysis showed that demographic composition was 29.4% associated with premature stroke mortality; socioeconomic status, 19.6%, health care and environmental features, 22.7%; and population health, 28.2% (Table 3).

**Table 3.** County-Level Factors Associated With Mortality per 100 000 Population Due to Stroke in US Residents by Place of Death, 2011-2018^a^

County characteristic	Total		Out-of-stroke-unit		In-hospital	
	Standard dominance statistic	Coefficient (95% CI)	Standard dominance statistic	Coefficient (95% CI)	Standard dominance statistic	Coefficient (95% CI)
Demographic composition, %						
Rural	0.198 (29.4)	0.001 (−0.001 to 0.001)	0.194 (31.6)	0.002 (−0.001 to 0.004)^b^	0.192 (28.7)	0.002 (0.001 to 0.003)^c^
Women		0.019 (0.012 to 0.025)^c^		−0.001 (−0.022 to 0.020)		0.005 (−0.005 to 0.016)
Age >64 y		0.012 (0.008 to 0.016)^c^		0.022 (0.012 to 0.031)^c^		0.010 (0.004 to 0.016)^c^
Black/African American		0.007 (0.006 to 0.008)^c^		0.006 (0.003 to 0.009)^c^		0.007 (0.005 to 0.009)^c^
American Indian/Alaskan Native		−0.005 (−0.007 to −0.002)^c^		−0.023 (−0.032 to −0.015)^c^		0.001 (−0.003 to 0.004)
Asian		0.022 (0.016 to 0.029)^c^		0.021 (0.007 to 0.035)^c^		0.023 (0.015 to 0.031)^c^
Born outside the US, %		−0.017 (−0.021 to −0.012)^c^		−0.027 (−0.038 to −0.017)^c^		−0.013 (−0.019 to −0.007)^c^
Socioeconomic status						
Median household income, $1000	0.132 (19.6)	−0.009 (−0.011 to −0.007)^c^	0.109 (17.7)	−0.010 (−0.015 to −0.005)^c^	0.125 (18.7)	−0.008 (−0.011 to −0.005)^c^
Unemployed		0.004 (−0.005 to 0.013)		0.008 (−0.015 to 0.031)		0.013 (0.001 to 0.025)^d^
Uninsured rate		0.019 (0.015 to 0.023)^c^		0.032 (0.022 to 0.042)^c^		0.013 (0.007 to 0.018)^c^
Health care and environmental features						
Primary care physicians per 100 000 population	0.153 (22.7)	−0.001 (−0.002 to −0.001)^d^	0.159 (25.8)	−0.001 (−0.001 to 0.001)	0.152 (22.7)	−0.001 (−0.001 to 0.001)
Health care quality index		−0.110 (−0.140 to −0.076)^c^		−0.220 (−0.300 to −0.130)^c^		−0.078 (−0.130 to −0.031)^c^
Access to exercise opportunities, %		−0.001 (−0.002 to −0.001)^d^		−0.003 (−0.005 to −0.001)^b^		−0.001 (−0.002 to 0.001)
Food environment index		0.013 (−0.008 to 0.033)		−0.008 (−0.058 to 0.043)		0.007 (−0.022 to 0.036)
Population health, %						
Physical inactivity	0.190 (28.2)	0.012 (0.007 to 0.017)^c^	0.153 (24.9)	0.020 (0.007 to 0.032)^c^	0.199 (29.8)	0.011 (0.005 to 0.018)^c^
Diabetes		0.019 (0.006 to 0.033)^c^		−0.001 (−0.037 to 0.036)		0.023 (0.004 to 0.042)^d^
Self-reported poor health status		0.005 (0.001 to 0.009)^d^		−0.002 (−0.015 to 0.011)		0.012 (0.006 to 0.019)^c^
Total Medicare reimbursements per enrollee, $1000		0.016 (0.004 to 0.027)^c^		0.022 (−0.006 to 0.050)		0.003 (−0.013 to 0.019)
Adjusted *R*^2^	NA	0.671 (NA)	NA	0.609 (NA)	NA	0.665 (NA)

Abbreviation: NA, not applicable.

^a^ Data on mortality from Centers for Disease Control and Prevention WONDER and data on county characteristics from the multiple county-level databases in 2011-2018 were combined. Dominance statistics can be used as an index of effect size and were derived as a weighted average marginal/incremental contribution to the overall fit statistic that an independent variable makes across all models in which the independent variable is included. The percentage of dominance statistics can describe the contribution of the 4 sets of factors associated with premature stroke mortality.

^b^ *P* < .10.

^c^ *P* < .01.

^d^ *P* < .05.

For out-of-stroke-unit death, county-level premature stroke mortality was associated with demographic composition (31.6%) and health care and environmental features (25.8%). For in-hospital death, 29.8% of county-level mortality was accounted for by population health and 28.7% was noted for demographic composition. Percentage of rural residents, percentage of those older than 64 years, percentage of Black or African American, percentage of Asian, uninsured rate, and prevalence of physical inactivity were positively associated with both out-of-stroke-unit and in-hospital death rates. Percentage of individuals born outside the US, income, and health care quality index were negatively associated with mortality for both out-of-stroke-unit and in-hospital mortality. For out-of-stroke-unit death, each 1-point increase in the percentage of American Indian or Alaska Native individuals’ mortality was lower by 0.023 (95% CI, −0.032 to −0.015) deaths per 100 000 population. For in-hospital death, the prevalence of diabetes and the self-reported poor health status were associated with mortality at 0.023 (95% CI, 0.004 to 0.042) and 0.012 (95% CI, 0.006 to 0.019) deaths per 100 000 population (Table 3).

Factors associated with county-level mortality also varied by stroke subtypes (eTable 2 in the Supplement). For each of the subtypes, premature stroke mortality was associated with demographic composition. The percentage of rural residents was positively associated with mortality of each subtype. The percentage of American Indian or Alaskan Native individuals, the percentage of individuals born outside the US, median household income, and health care quality index were negatively associated with mortality for both intracerebral hemorrhage and those not specified as hemorrhage or infarction. For those not specified as hemorrhage or infarction, the number of deaths per 100 000 population was associated with the following factors: the percentage of women (0.018; 95% CI −0.002 to 0.0037), the percentage of those older than 64 years (0.014; 95% CI, 0.004 to 0.023), the percentage of Black or African American individuals (0.007; 95% CI, 0.004 to 0.010), the percentage of Asian individuals (0.020; 95% CI, 0.006 to 0.035), the uninsured rate (0.021; 95% CI, 0.012 to 0.031), and the prevalence of physical inactivity (0.026; 95% CI, 0.015 to 0.038). For other and sequelae of cerebrovascular disease, mortality association for the percentage of American Indian or Alaskan Native individuals, the percentage of individuals born outside the US, and food environment index were −0.028 (95% CI, −0.056 to −0.001), −0.028 (95% CI, −0.052 to −0.003), and −0.200 (95% CI, −0.370 to −0.031) deaths per 100 000 population.

## Discussion

The findings of this study provide insight into which features may predispose certain counties to stroke mortality disadvantage. We highlighted the large percentage reporting stroke not specified as hemorrhage or infarction, with the majority of deaths occurring out of the stroke unit. We further reported the heterogeneity of stroke subtypes, with the highest between-county disparities found for those not specified as hemorrhage or infarction and other and sequelae of cerebrovascular disease. Our study also illustrates the considerable differences in place of death and stroke subtype in terms of between-county disparity and its associated factors.

Our findings are consistent with a study that found a high percentage of certificates, which complicates the comparability of stroke subtype mortality.^5^ Although patients with hemorrhagic stroke were reported to have higher overall mortality than ischemic stroke,^20^ one study pointed out that most physicians tend not to be taught to report cause-of-death statements specifically, and the querying for unspecified cause-of-death statements varied by state.^5^ Our study adds the value that the uncertainty of the cause-of-death statements were accounted for largely by within-state variations. Our findings also show that counties with higher percentages of rural, female, Black or African American, Asian, and uninsured populations were more likely to have higher mortality of stroke causes not specified as hemorrhage or infarction.

We further used place of death to better clarify the possible factors associated with between-county disparities in cause-specific mortality. We found that most of the strokes not specified as hemorrhage or infarction and other and sequelae of cerebrovascular disease occurred out of a stroke unit. Although stroke is frequently nonlethal, 27.96% of deaths occurred out of a stroke unit, which may occur because of unexpectedly sudden death related to problems of delays in seeking health care or coexisting conditions (eg, cancer, cardiovascular disease) during rehabilitation. It is difficult to identify a stroke death and trace back the specific information on the stroke subtype. Therefore, it is necessary to develop plans to mitigate the effect of such uncertainty to improve the quality of stroke subtype mortality data for documentation of out-of-stroke-unit death on the death certificate.

The rate of premature stroke mortality did not change substantially during the study period, with the percentage of out-of-stroke-unit deaths increasing by 11 percentage points (23.56% in 1999, 34.57% in 2018). An earlier study found similar patterns of geographic disparities in stroke incidence and stroke mortality, but the magnitude of disparities in mortality across counties appears larger than that of the incidence.^21^ This difference may indicate that disparities in case fatality (eg, health risk profile and access to quality health care) can be major contributors to mortality disparities. Our findings on the association between mortality and 4 sets of county-level factors identified potentially modifiable county-level health care delivery and risk factors worthy of policy makers’ attention.

Our study identified demographic composition associated with mortality for both out-of-stroke-unit and in-hospital deaths. Some studies have shown race and sex disparities in stroke mortality, which can be partially associated with the disparities throughout the continuum of care,^22,23^ including access to and quality of care,^24,25,26^ and identification and control of risk factors (eg, hypertension, access to physician care, and medications).^27,28^ Some studies also suggest that these mortality disparities can be associated with regional-level (eg, access to and activation of emergency medical services)^29,30^ or system-related (eg, hospital characteristics) factors.^31,32^ In many countries, evidence shows that people with lower socioeconomic status are less likely to receive good-quality acute hospital and rehabilitation care than people with higher socioeconomic status.^33,34^ For clinical practice, better implementation of evidence-based interventions, including management of risk factors, and access to high-quality stroke care and rehabilitation may reduce these disparities.

Our findings also suggest that health care and environmental factors were associated more frequently with out-of-stroke unit mortality than with in-hospital mortality, and population health more with in-hospital mortality than with out-of-stroke unit mortality. The explanation for these associations is probably multifactorial, and stroke subtype may contribute to a better understanding of the findings. Hemorrhagic stroke is one of the acute cerebrovascular events and is more likely to be associated with out-of-stroke-unit death than other subtypes of stroke. Hemorrhagic stroke requires rapid coordination of care beginning at the time of symptom onset, with high requirements for improving the quality of health care. Meanwhile, our study noted that the health care quality index was negatively associated with mortality due to intracerebral hemorrhage. Previous studies also reported that groups with less access to and low quality of health care experience more severe strokes with higher acute-phase case fatality rates, indicating more disparities in acute stroke treatment for those that occurred out of the stroke unit.^35^ Therefore, health care and environmental features were associated more frequently with out-of-stroke unit mortality than in-hospital mortality. Patients diagnosed with cerebral infarction who have a greater burden of vascular risk factors and comorbidity have the worse prognosis in the subacute phase of stroke and have higher subacute case fatality rates, most often with in-hospital stroke.^35^ Thus, population health is associated with more in-hospital mortality than out-of-stroke-unit mortality.

### Limitations

This study had several limitations. First, the retrospective cross-sectional analysis limited the ability to draw any causal inference from the findings. In addition, as ecologic research with county-level databases, this study may have ecologic fallacy. Thus, *R*^2^ values for ecologic studies are typically much higher than in individual-level studies. Thus, our results need to be interpreted with caution. Second, approximately one-third of deaths were not specified as hemorrhage or infarction, and the county-level variation was large, which may hinder the comparability of stroke subtype mortality across counties. Third, there may be some other contributory factors not considered in this analysis that vary across counties. One such factor in county-level variation is health policies for prevention and control of stroke. Fourth, the prevalence of hypertension and hypercholesterolemia may be associated with stroke mortality, but these variables were not included in this study owing to data limitations. The lack of associated variables may undermine the accuracy of dominance analysis results. Fifth, the time of onset of disease symptoms and exact time of death were not available for analyses, which limited our ability to identify the first episode or relapse, which may have different patterns of mortality and between-county disparities.

## Conclusions

Premature stroke mortality noted in this study decreased from 1999 to 2018, but the between-county disparity still exists. This study serves to suggest that strategies should address specific factors that underlie the mortality disparities, especially for the out-of-stroke-unit deaths and stroke of uncertain cause and be tailored to local context before implementing interventions for the neediest counties. Our results also suggest there is a need to improve the quality of stroke subtype mortality data for documentation of out-of-stroke-unit death on the death certificate.

## Notes

Supplement. **eTable 1.** Variables Included in the Regression Analysis With Data Source and Summary Statistics **eTable 2.** County-Level Factors Associated With Mortality (per 100 000 Population) Due to Stroke in the US Residents Aged 25-64 Years by Subtype, 2011-2018 **eFigure.** Trends in Crude County-Level Mortality (per 100 000 Population) Due to Stroke, and Distribution of Place of Death and Subtype in US Residents Aged 25-64 years, 1999-2018
