# Supplementary material for: Evaluation of Between-County Disparities in Premature Mortality Due to Stroke in the US
Source: JAMA Netw Open. 2021 May 12;4(5):e214488. doi: 10.1001/jamanetworkopen.2021.4488 (PMC8116984; doi:10.1001/jamanetworkopen.2021.4488)
Supplement: Supplement. — eTable 1. Variables Included in the Regression Analysis With Data Source and Summary Statistics eTable 2. County-Level Factors Associated With Mortality (per 100 000 Population) Due to Stroke in the US Residents Aged 25-64 Years by Subtype, 2011-2018 eFigure. Trends in Crude County-Level Mortality (per 100 000 Population) Due to Stroke, and Distribution of Place of Death and Subtype in US Residents Aged 25-64 years, 1999-2018 [file jamanetwopen-e214488-s001.pdf]

**Supplementary Online Content**

Song S, Ma G, Trisolini MG, et al. Evaluation of between-county disparities in premature mortality due to stroke in the US. *JAMA Netw Open*. 2021;4(5):e214488. doi:10.1001/jamanetworkopen.2021.4488

**eTable 1.** Variables Included in the Regression Analysis With Data Source and Summary Statistics

**eTable 2.** County-Level Factors Associated With Mortality (per 100,000 Population) Due to Stroke in the US Residents Aged 25-64 Years by Subtype, 2011-2018

**eFigure.** Trends in Crude County-Level Mortality (per 100,000 population) Due to Stroke, and Distribution of Place of Death and subtype in the US Residents Aged 25-64 years, 1999-2018

This supplementary material has been provided by the authors to give readers additional information about their work.

© 2021 Song S et al. *JAMA Network Open.*

**eTable 1** Variables included in the regression analysis with data source and summary statistics

| County Characteristics | Data source | Median (Interquartile ranges) |
|---|---|---|
| Demographic composition | | |
| Rural, %, 2011-2017[g] | CHRR[a] | 48.04 (24.25, 69.18) |
| Female, %, 2011–2017[g] | CPE[b] | 50.63 (49.97, 51.25) |
| Aged >64 years, %, 2011–2017[g] | CPE[b] | 16.44 (14.29, 18.68) |
| Black/African American, %, 2011–2017[g] | CPE[b] | 4.34 (1.19, 15.38) |
| American Indian/Alaskan Native, %, 2011–2017[g] | CPE[b] | 0.52 (0.33, 1.01) |
| Asian, %, 2011–2017[g] | CPE[b] | 0.82 (0.50, 1.69) |
| Born outside the US, %, 2011–2017[g] | ACS[c] | 3.14 (1.55, 6.20) |
| Socioeconomic status | | |
| Median household income, $1000, 2011-2018 | SAIPE[d] | 45.94 (39.74, 54.41) |
| Unemployed, %, 2011–2017[g] | CHRR[a] | 6.39 (5.41, 7.48) |
| Uninsured rate, %, 2011-2017[g] | SAHIE[e] | 13.33 (10.19, 16.81) |
| Health care and environmental features | | |
| Primary care physicians, per 100,000 population, 2011-2018 | CHRR[a] | 52.46 (36.88, 74.27) |
| Healthcare quality index, 2011-2015[g,h] | DAHC[f] | 0.11 (-0.15, 0.36) |
| Access to exercise opportunity, %, 2012-2018[g,i] | CHRR[a] | 65.10 (50.23, 79.04) |
| Food environment index, 2011-2016[g,j,k] | CHRR[a] | 7.48 (6.77, 8.03) |
| Population health | | |
| Physical inactivity, %, 2011-2018 | CHRR[a] | 27.46 (23.76, 30.86) |
| Diabetes, %, 2011-2018 | CHRR[a] | 10.92 (9.44, 12.44) |
| Self-reported poor health status, %, 2011-2018 | CHRR[a] | 17.31 (13.94, 21.12) |
| Total Medicare reimbursements per enrollee, $1000, 2011-2016[g] | DAHC[f] | 9.80 (8.94, 10.67) |

Abbreviations: ACS, American Community Survey; CHRR, County Health Rankings & Roadmaps; CPE, Census Population Estimates; DAHC, Dartmouth Atlas of Health Care; SAHIE, Small Area Health Insurance Estimates; SAIPE, Small Area Income and Poverty Estimates.

[a]CHRR database provides a model to help communities understand the factors influencing healthy residents. It summaries many health outcome and health factors from other databases each year. Available at: http://www.countyhealthrankings.org Accessed 06/20/2019.

[b]Available at:https://factfinder.census.gov/faces/nav/jsf/pages/searchresults.xhtml?refresh=t Accessed 06/20/2019.

[c]ACS, 5-year estimates. Available at:http://factfinder.census.gov/faces/nav/jsf/pages/download_center.xhtml Accessed 06/20/2019.

[d] Available at:https://www.census.gov/programs-surveys/saipe/data/datasets.html Accessed 06/20/2019.

[e] Available at:https://www.census.gov/programs-surveys/sahie.html Accessed 06/20/2019.

[f]Available at:http://www.dartmouthatlas.org/downloads/tables/ Accessed 06/20/2019.

[g]For those variables restricted to year range, we conducted ordinary least square regression model to predict the missing value.

[h]Calculated by taking the mean of the z-scores for six variables related to primary care access and quality among Medicare enrollees: the percent of enrollees who had an ambulatory care visit to a primary care clinician, the percent of diabetic enrollees who had an HbA1c test/an eye exam/an LDL-C test, the percent of female enrollees who had a mammogram; and the discharge rate for ambulatory care sensitive conditions.

© 2021 Song S et al. *JAMA Network Open.*

[i]The 2014-2019 CHRR databases summarized this variable from OneSource Global Business Browser, DeLorme map data, Esri, and Census Bureau TIGER/Line files from 2012-2018.
[j]The 2014-2019 CHRR were used to summarize this variable from US Department of Agriculture Food Environment Atlas, Map the Meal Gap from 2011-2016.
[k]Calculated as a composite score, ranging from 1 to 10, describing limits on access to healthy foods, with 1 indicating the lowest and 10 indicating the highest access to healthy foods.

© 2021 Song S et al. *JAMA Network Open.*

**eTable 2.** County-level factors associated with mortality (per 100,000 population) due to stroke in the US residents aged 25-64 years by subtype, 2011-2018

| County characteristics | Subarachnoid Hemorrhage | | Intracerebral Hemorrhage | | Cerebral Infarction | | Stroke, Not Specified as Hemorrhage or Infarction | | Other and Sequelae of Cerebrovascular Disease | |
|---|---|---|---|---|---|---|---|---|---|---|
| | Standard Dominance Statistic | Coefficient (95% CI) | Standard Dominance Statistic | Coefficient (95% CI) | Standard Dominance Statistic | Coefficient (95% CI) | Standard Dominance Statistic | Coefficient (95% CI) | Standard Dominance Statistic | Coefficient (95% CI) |
| **Demographic composition** | | | | | | | | | | |
| Rural, % | 0.146 (33.6%) | 0.007*** (-0.002, -0.016) | 0.221 (33.5%) | 0.002* (0.000, 0.004) | 0.260 (38.8%) | 0.013** (0.0004, 0.025) | 0.209 (30.6%) | 0.003*** (0.001, 0.005) | 0.159 (37.9%) | 0.013*** (0.006, 0.020) |
| Female, % | | -0.013 (-0.120, 0.091) | | -0.006 (-0.026, 0.015) | | -0.11 (-0.250, 0.036) | | 0.018* (-0.002, 0.037) | | -0.041 (-0.120, 0.034) |
| Aged >64 years, % | | 0.016 (-0.011, 0.043) | | 0.010* (-0.001, 0.021) | | 0.012 (-0.029, 0.053) | | 0.014*** (0.004, 0.023) | | 0.014 (-0.015, 0.043) |
| Black/African American, % | | 0.004 (-0.007, 0.014) | | 0.009*** (0.006, 0.012) | | 0.001 (-0.013, 0.015) | | 0.007*** (0.004, 0.010) | | 0.001 (-0.008, 0.010) |
| American Indian/Alaskan Native, % | | -0.006 (-0.028, 0.017) | | -0.013*** (-0.021, -0.005) | | -0.045 (-0.180, 0.089) | | -0.009** (-0.016, -0.001) | | -0.028** (-0.056, -0.001) |
| Asian, % | | 0.013 (-0.013, 0.038) | | 0.028*** (0.016, 0.040) | | 0.003 (-0.035, 0.041) | | 0.020*** (0.006, 0.035) | | 0.019 (-0.011, 0.048) |
| Foreign born, % | | -0.008 (-0.032, 0.015) | | -0.019*** (-0.029, -0.010) | | -0.016 (-0.050, 0.019) | | -0.024*** (-0.034, -0.015) | | -0.028** (-0.052, -0.003) |

© 2021 Song S et al. *JAMA Network Open.*

| | | | | | | | | | | | | | | |
|---|---|---|---|---|---|---|---|---|---|---|---|---|---|---|
| **Socioeconomic status** | | | | | | | | | | | | | | |
| Median household income, $1000 | 0.079 (18.3%) | -0.006 (-0.017, 0.005) | | 0.109 (16.5%) | -0.007*** (-0.012, -0.002) | | 0.089 (13.2%) | -0.004 (-0.023, 0.016) | | 0.111 (16.3%) | -0.010*** (-0.015, -0.006) | | 0.059 (14.1%) | 0.001 (-0.012, 0.014) |
| Unemployed, % | | 0.007 (-0.057, 0.071) | | | 0.022* (-0.001, 0.045) | | | 0.004 (-0.110, 0.120) | | | -0.002 (-0.024, 0.019) | | | -0.015 (-0.074, 0.044) |
| Uninsured rate, % | | 0.007 (-0.021, 0.035) | | | 0.020*** (0.009, 0.030) | | | 0.019 (-0.024, 0.063) | | | 0.021*** (0.012, 0.031) | | | 0.014 (-0.014, 0.041) |
| **Health care and environmental features** | | | | | | | | | | | | | | |
| Primary care physicians per 100,000 population | 0.100 (23.1%) | -0.001 (-0.003, 0.003) | | 0.150 (22.7%) | 0.001 (-0.001, 0.001) | | 0.170 (25.4%) | 0.001 (-0.004, 0.005) | | 0.172 (25.2%) | -0.001 (-0.002, 0.001) | | 0.104 (24.7%) | 0.001 (-0.002, 0.004) |
| Healthcare quality index | | -0.077 (-0.300, 0.150) | | | -0.130*** (-0.220, -0.045) | | | -0.069 (-0.440, 0.310) | | | -0.130*** (-0.210, -0.051) | | | -0.051 (-0.280, 0.170) |
| Access to exercise opportunities, % | | -0.001 (-0.012, 0.010) | | | -0.002 (-0.005, 0.001) | | | 0.002 (-0.014, 0.018) | | | -0.001 (-0.003, 0.002) | | | 0.007 (-0.003, 0.017) |
| Food environment index | | 0.053 (-0.140, 0.250) | | | 0.036 (-0.019, 0.092) | | | -0.084 (-0.360, 0.190) | | | -0.012 (-0.058, 0.034) | | | -0.200** (-0.370, -0.031) |
| **Population health** | | | | | | | | | | | | | | |
| Physical inactivity, % | 0.109 (25.1%) | 0.014 (-0.024, 0.052) | | 0.180 (27.3%) | 0.003 (-0.010, 0.015) | | 0.152 (22.6%) | 0.016 (-0.041, 0.073) | | 0.189 (27.8%) | 0.026*** (0.015, 0.038) | | 0.099 (23.4%) | 0.007 (-0.030, 0.044) |
| Diabetes, % | | -0.022 (-0.130, 0.090) | | | 0.012 (-0.024, 0.049) | | | 0.048 (-0.110, 0.210) | | | 0.003 (-0.030, 0.037) | | | -0.004 (-0.110, 0.100) |
| Self-reported poor health status, % | | 0.018 (-0.026, 0.063) | | | 0.022*** (0.009, 0.035) | | | 0.018 (-0.048, 0.085) | | | 0.007 (-0.005, 0.019) | | | 0.055*** (0.015, 0.096) |
| Total Medicare reimbursements per enrollee, | | -0.037 (-0.130, 0.054) | | | -0.004 (-0.034, 0.026) | | | -0.064 (-0.200, 0.073) | | | 0.017 (-0.009, 0.043) | | | -0.008 (-0.084, 0.069) |

© 2021 Song S et al. *JAMA Network Open.*

| | | | | | | | | | | | | | | |
|---|---|---|---|---|---|---|---|---|---|---|---|---|---|---|
| $1,000 | | | | | | | | | | | | | | |
| **Adjusted $R^2$** | 0.410 | | | 0.654 | | | 0.649 | | | 0.675 | | | 0.395 | |

*Note:* We combined the data on mortality from CDC wonder and data on county characteristics from the multiple county-level databases in 2011-2018.

Dominance statistics can be used as an index of effect size, and are derived as a weighted average marginal/incremental contribution to the overall fit statistic an independent variable makes across all models in which the independent variable is included. The percent of dominance statistics can describe the contribution of the four sets of factors associated with the premature stroke mortality.

*** P<0.01, ** P<0.05, * P<0.10

© 2021 Song S et al. *JAMA Network Open.*

**eFigure.** Trends in crude county-level mortality (per 100,000 population) due to stroke, and distribution of place of death and subtype in the US residents aged 25-64 years, 1999-2018.

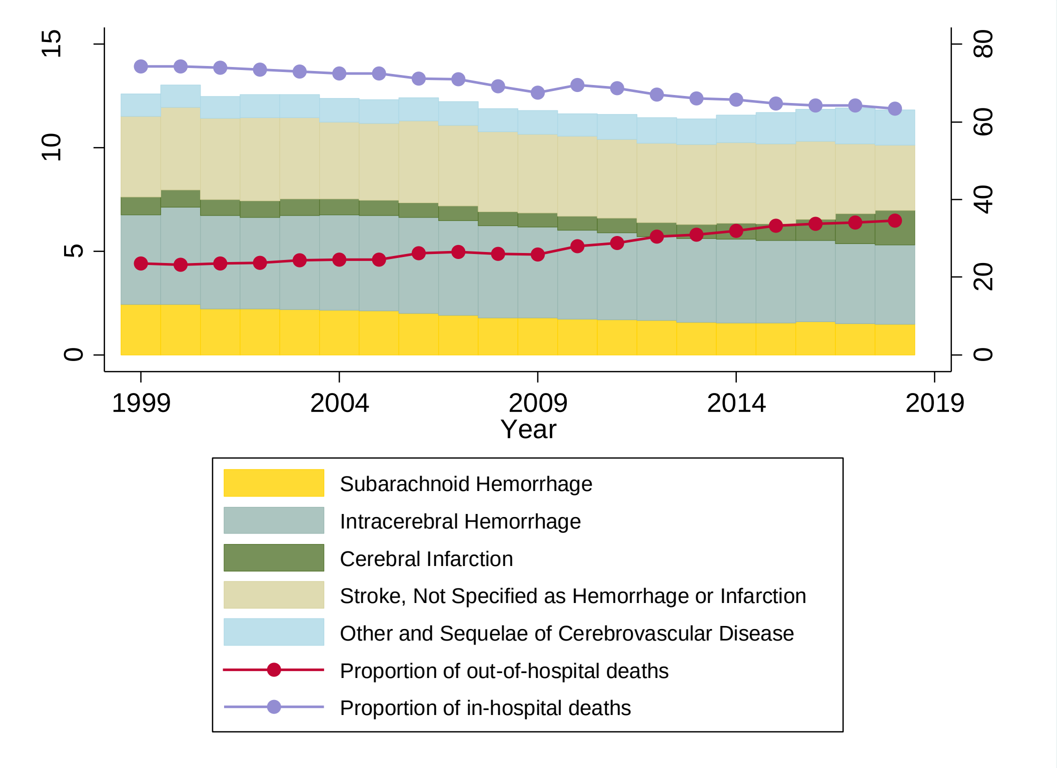


© 2021 Song S et al. *JAMA Network Open.*
